# Antimicrobial properties of WCO-based composites enriched with hops and curly sorrel for green building solutions

**DOI:** 10.1371/journal.pone.0307452

**Published:** 2024-07-18

**Authors:** Anita Staroń, Barbara Pucelik, Agata Barzowska, Magda Kijania-Kontak, Paweł Staroń

**Affiliations:** 1 Department of Engineering and Chemical Technology, Cracow University of Technology, Cracow, Poland; 2 Malopolska Centre of Biotechnology, Jagiellonian University, Cracow, Poland; 3 Doctoral School of Exact and Natural Sciences, Jagiellonian University, Cracow, Poland; 4 Department of Civil Engineering, Cracow University of Technology, Cracow, Poland; Luleå University of Technology, SWEDEN

## Abstract

Modern production of vegetable oils has reached impressive levels, and the ever-growing quantities of waste cooking oil (WCO) provide a local source of raw materials for innovative materials. The WCO composite production process involves a series of reactions, including polymerisation, esterification, and transesterification, which lead to the hardening of composite materials. In light of the growing problem of bacterial and fungal diseases, materials with high strength properties and biocidal properties are being sought. Fungal infections of the skin are a widespread problem, and the number of cases is steadily increasing. This article presents a study of the antibacterial potential of WCO-based composites enriched with hops or sorrel root in the context of their application in the construction industry. The compressive and flexural strength of the oil composites, their absorbability and hydrophobicity, and their effects on Gram-positive (*S*. *aureus* and *S*. *epidermidis*) and Gram-negative (*E*. *coli* and *P*. *aeruginosa*) bacteria and fungi (*A*. *niger*, *P*. *anomala*) were investigated. Maximum split tensile strength (4.3 MPa) and flexural strength (5.1 MPa) were recorded for oil-hop composites. Oil composites enriched with curly sorrel and hops showed antibacterial activity against *S*. *aureus* at 27% and 25%. High biocidal activity (up to 70%) was recorded against *E*. *coli* and against *S*. *epidermidis* (up to 99%) due to the action of composites with curly sorrel. The antifungal activities of composites with hops was 15% and 19% for *P*. *anomala* and *A*. *niger*, respectively, while with curly sorrel they were 42% and 30%.

## Introduction

Composites based on waste cooking oil (WCO) can find applications in the construction industry, including roofing tiles, paving blocks, sidewalk edges, and pavement slabs [1]. These materials, commonly referred to as "green materials," are produced using waste generated from the food frying process. In the years 2021–2022, global production of vegetable oils reached nearly 210 million tons [2]. The quantity of produced WCO continues to grow annually, making it a locally available raw material source [3, 4]. The production process involves the use of an acidic catalyst, such as sulfuric acid, to expedite the solidification of rapeseed oil after the frying process. Additionally, sand is introduced into the mixture as an aggregate material.

The solidification of the oil is a result of a series of reactions that occur during the process. The initial reaction is polymerisation, followed by esterification. During esterification, the diols and diacids present in the oil after frying undergo reactions, resulting in the formation of polyesters that harden the matrix of the blocks [5, 6]. The transesterification process reduces the number of acidic compounds in the frying oil, where the fatty acids contained in it react with alkyl alcohols (e.g., methanol) in the presence of alkyl catalysts (e.g., sodium or potassium hydroxide). An additional product generated is glycerol, which may exist in the form of a mixture with the catalyst, non-degradable fatty acids, alcohol, or other impurities. In the initial stage of transesterification, the base reacts with the alcohol, forming an alcoholate and a catalyst with protons. In the second step, the alcoholate initiates a nucleophilic attack on the carbonyl group of the triglyceride, creating an intermediate product. The resulting anions of diglycerides react with the catalyst with protons, generating a pure catalyst capable of commencing further transformations. The resulting diglycerides and monoglycerides are further transformed into a mixture of esters and glycerol [7, 8]. In the process of obtaining WCO-based composite materials, Brønsted acids are also used, most often sulfuric acid (VI) due to its high transesterification efficiency [9]. During the heating process, the compounds contained in WCO undergo polyesterification, creating solid polyester. Diols present in waste cooking oil along with dicarboxylic acids participate in the polyesterification reaction, leading to the formation of solid polyesters, which serve as the matrix for blocks. Polymerization reaction can also proceed via a different mechanism, in which after the formation of a carbocation, there is no attack on the double bond. Instead, the carbocation with a hydrogen atom is shifted from a monoallylic or bis-allylic position. This occurs because allylic cations exhibit lower reactivity than carbocations formed by proton attachment to a double bond. The final product can be polyester, water, and regenerated acid [10]. In the absence of an acidic catalyst in the system, thermal processes within the vegetable oil initiate a polymerisation reaction involving oxygen, increasing the oil’s viscosity and causing it to harden [11]. Undoubtedly, the factors determining the utilisation of oil-based composites as construction materials include the ability to source raw materials locally, the durability of the composites, their capacity to be used in accordance with their properties, and their life cycle.

Due to the rapid spread of bacterial and fungal diseases, there is a search for materials with high durability parameters while simultaneously exhibiting biocidal properties. These infections are a consequence of changes in social, demographic, economic, and, most importantly, health-related indicators, impacting the quality and comfort of people’s lives. Advances in the field of medicine have reassured people and alleviated a longstanding issue, leading to the emergence of infectious diseases on a larger scale. Many infectious diseases are transmitted through social contacts, and the current increase in social connectivity undeniably accelerates the transmission dynamics. Superficial fungal infections are prevalent worldwide, affecting an estimated 20–25% of the global population, with the incidence of superficial fungal infections continuing to rise [12, 13]. The challenge, therefore, is to obtain functional composite materials based on waste cooking oil containing natural additives for applications in places and public facilities with a higher risk of pathogens. In the present study, curly dock (*Rumex crispus L*.) and common hop (*Humulus lupulus*) were used as raw materials with antimicrobial potential.

Curly dock (*Rumex crispus* L.), also known as horse sorrel, exhibits a high level of antibacterial effectiveness. Curly dock is considered by farmers to be a troublesome weed that overgrows meadows and pastures. In the literature, numerous pieces of information can be found regarding the antimicrobial properties of curly dock against *Escherichia coli*, *Proteus mirabilis*, *Candida spp*., *Pseudomonas aeruginosa*, and *Trichophyton mentagrophytes* [14]. Extracts from curly dock also show inhibitory effects on staphylococci, including pathogenic *S*. *aureus* and opportunistic *S*. *epidermidis*, as well as on certain Gram-negative bacteria, such as *P*. *vulgaris*. Additionally, antifungal effects of curly dock have been observed against yeast strains like *Candida albicans* and dermatophytes such as *Trichophyton mentagrophytes*, as well as against *Acinetobacter baumannii* from wounds, including multidrug-resistant strains, using alcoholic extracts of curly dock [14, 15]. Antibacterial activity against bacteria has also been observed for aqueous and acetone extracts of curly dock, which is why they are used in dermatology [16, 17]. These extracts also possess additional biological properties, including anticancer, antioxidant, antiviral, anti-scurvy, immunostimulatory, and antipyretic effects [18, 19].

Among natural sources, common hop (*Humulus lupulus*) is also popular. Most hop cultivation is used in the brewing industry due to its bitter taste, stemming from the presence of hydrophobic compounds (bitter α and β acids) in hop cones, as well as its characteristic hoppy aroma (essential oils) [20]. Hop bracts, which are not utilised in beer production, represent waste materials that possess valuable properties [21]. The components of hops exhibit biocidal activity against Gram-positive bacterial species, including *Lactobacillus*, *Staphylococcus*, *Streptococcus*, *Bacillus*, *Micrococcus*, *Pediococcus*, and *Actinomyces naeslundii*, as well as Gram-negative bacteria such as *Prevotella oralis*, *Prevotella melaninogenica*, *Fusobacterium nucleatum*, *Chlamydia pneumonia*, *Escherichia coli*, and protozoa [22–24].

The aim of this research was to produce composites containing hops or curly dock root and to evaluate their antimicrobial properties against selected Gram-positive bacteria, Gram-negative bacteria and fungi. The results of the research will determine the effect of catalysed waste cooking oil and the annealing process on the properties of the natural additive, and thus on the biocidal efficacy of the composites.

## Materials and methods

### Preparation of functional composites enriched with hops or curly sorrel

The composites were obtained by annealing a mixture of waste cooking oil (WCO), sulfuric acid VI (pure for analysis), aggregate, and a natural additive: hops (H) or curly sorrel root (CS). WCO was rapeseed oil after the food frying process; air-dry quartz sand with a fraction size of 0.5–1.4 mm was used as aggregate. Natural additives were purchased from a herbal store in dried form. Using a planetary mixer, WCO was mixed with sulfuric acid first, and then sand was added. After 5 minutes of homogenisation, dried herbs were added to the mixture and homogenised again. The material was compacted in aluminum moulds on a vibrating table and then annealed at 190 to 210°C for 12–20 hours. The content of catalysed oil and dried plant matter was counted relative to the weight of the sand. A total of 100 materials, each enriched with hops or curly sorrel root, were obtained, but only composites meeting the strength requirements for paving blocks were selected for testing antimicrobial properties. Selected composites and the parameters of the process of obtaining these composites are presented in Table 1.

**Table 1:** Parameters of composite materials with the natural additive production process.

Composite No.	Catalysed oil [%]	H_2_SO_4_/catalysed oil [g/g]	Temperature [°C]	Time [h]	Additive [%]
12	25	0.24	190	20	1
16	25	0.24	210	20	7
22	25	0.14	200	19	4
35	25	0.24	210	18	1
41	20	0.24	210	20	1
43	25	0.24	210	20	7
44	22.5	0.14	200	18	4
51	22.5	0.24	200	19	4
85	22.5	0.14	210	18	7
86	22.5	0.14	210	19	7
88	22.5	0.24	210	19	7
92	22.5	0.24	210	19	1
99	22.5	0.24	210	12	4

### Characteristics of composites

Thermal analysis of the composites was carried out in the temperature range of 25–1000°C at a heating rate of 10°C/min using an SDT 650 from TA Instruments. Fourier transform infrared spectroscopy (wavelength range 400–4000 cm^-1^) was used to analyse the molecular structure of the composites using a Nicolet iS5 FT-IR spectrometer from Thermo Scientific. The surface structure of the materials was observed using a Hitachi TM-3000 scanning electron microscope (SEM) equipped with an energy dispersive X-ray microanalyser (EDS). The mechanical strength of the composites was determined using a Zwick-Roell Z600 testing machine with an initial force of 25 N and a testing speed of 1 kN/min. The soakability of the composites was determined by calculating the sample’s weight gain and the percentage of water content in the sample after the incubation period (72 hours at room temperature). Hydrophobicity was determined based on the contact angle formed between the surface and the tangent plane to the liquid surface (distilled water). A hydrophobic surface is characterised by a contact angle less than 90°.

### Cultivation of bacterial and fungal strains

In this research, we utilised both Gram-positive (*S*. *aureus* and *S*. *epidermidis*) and Gram-negative bacteria (*E*. *coli* and *P*. *aeruginosa*). The strains *S*. *aureus* (ATCC 8325), *S*. *epidermidis* (ATCC12228), *E*. *coli* (K12), and *P*. *aeruginosa* (ATCC19660) were propagated in brain heart infusion (BHI) broth or LB broth. Cultivation was carried out at 37°C with shaking (180 rpm) in an orbital incubator. The growth of these cells was monitored by measuring the optical density at 600 nm (OD600), targeting an absorbance of 0.5, which is equivalent to around 10^*7*^ CFU/mL.

In this study, strains of *P*. *anomala* and *A*. *niger* were used. Cultures of *P*. *anomala* were maintained on Sabouraud dextrose agar plates. These cultures were transferred to freshly prepared SDA plates and incubated at 30°C for at least 48 hours. For all planktonic and biofilm tests, yeast extract peptone dextrose broth, Sabouraud dextrose broth (SDB), and RPMI 1640 were used as culture media. *A*. *niger* was inoculated and cultured with shaking (100 rpm) in Czapek-peptone medium at 30°C for at least 7 days before the experiments.

### Biofilm development process

Overnight-grown *S*. *aureus* colonies were resuspended in appropriate media to an OD490 of 0.65. This bacterial suspension was then diluted in a 1:6 ratio and incubated at 37°C with 5% CO_*2*_ for about 3 hours to reach the mid-log phase. The suspension was further diluted (1:2500) and 200 μL was added to each well of an 8-well chamber slide coated with thin-layer agar. After 16 hours, the medium was replaced with fresh medium and the biofilm was treated with selected composites for 24 hours, followed by fluorescence microscopy visualisation.

### Evaluating the antibacterial properties of composite materials

The antibacterial and antifungal efficacy of various composites (concentrations ranging from 0 to 100 mg/mL) was tested against the bacteria and fungi in PBS. After 24 hours of incubation in the dark at room temperature, 10 μL aliquots were transferred to fresh growth media in a 12-well plate. These samples were serially diluted and plated on LB and BHI agar to determine CFU counts. Bacterial viability was assessed using the LIVE/DEAD BacLight bacterial viability kit, while the viability of fungi was monitored using XTT and Resazurin viability assays (Invitrogen), both following the manufacturer’s guidelines, which assess cell membrane integrity.

### Flow cytometry analysis

To further assess the antibacterial and antifungal activity of the composites, flow cytometry was employed. Bacteria and fungi were stained with propidium iodide (10 μg/mL) to detect dead cells through red fluorescence. Both control and treated bacteria and fungi (0.5 × 10^*6*^ cells) were stained, washed, and prepared for analysis. After centrifugation, bacteria and fungi were resuspended in 200 μL of PBS and analysed using a BD c6 Accuri flow cytometer. Data analysis was conducted using FlowJo (MerckMillipore, Burlington, MA, USA) and GraphpadPrism 5 software.

### Fluorescence microscopy for bacterial and fungal examination

The antibacterial impact of the materials was visualised using a Zeiss LSM880 confocal microscope. Post-treatment, bacteria, and fungi were stained with Calcein AM and propidium iodide. Live cells exhibited green fluorescence, while dead cells showed red fluorescence. The samples were then placed on glass slides for imaging and analysed using Zeiss ZEN software.

### Biofilm imaging technique

Biofilms in each chamber were washed and stained with BacLight Live/Dead stain, followed by incubation at room temperature for 15 minutes. After staining, biofilms were washed, fixed with neutral buffered formalin, and then washed again. The biofilms were then mounted and imaged using a Zeiss880 confocal microscope, with image analysis conducted using Zeiss ZEN software.

### Cytotoxicity assessment in human keratinocytes

The human keratinocyte cell line HaCaT was cultured in DMEM supplemented with additives. Post-confluence, cells were harvested and seeded in 96-well plates. After treatment with composite materials at 100 mg/mL for 24 hours, cell viability was assessed using AlamarBlue and MTT assays. The absorbance and fluorescence were measured, and cell morphology was examined using bright-field microscopy. Each experiment was replicated three times.

### Statistical methodology

Data are presented as mean ± standard deviation. Statistical significance was determined using one-way or two-way ANOVA with Bonferroni post hoc tests, utilising GraphPad Prism version 5.0.0.

## Results and discussion

### Oil composites characterisation

**Fig 1:**
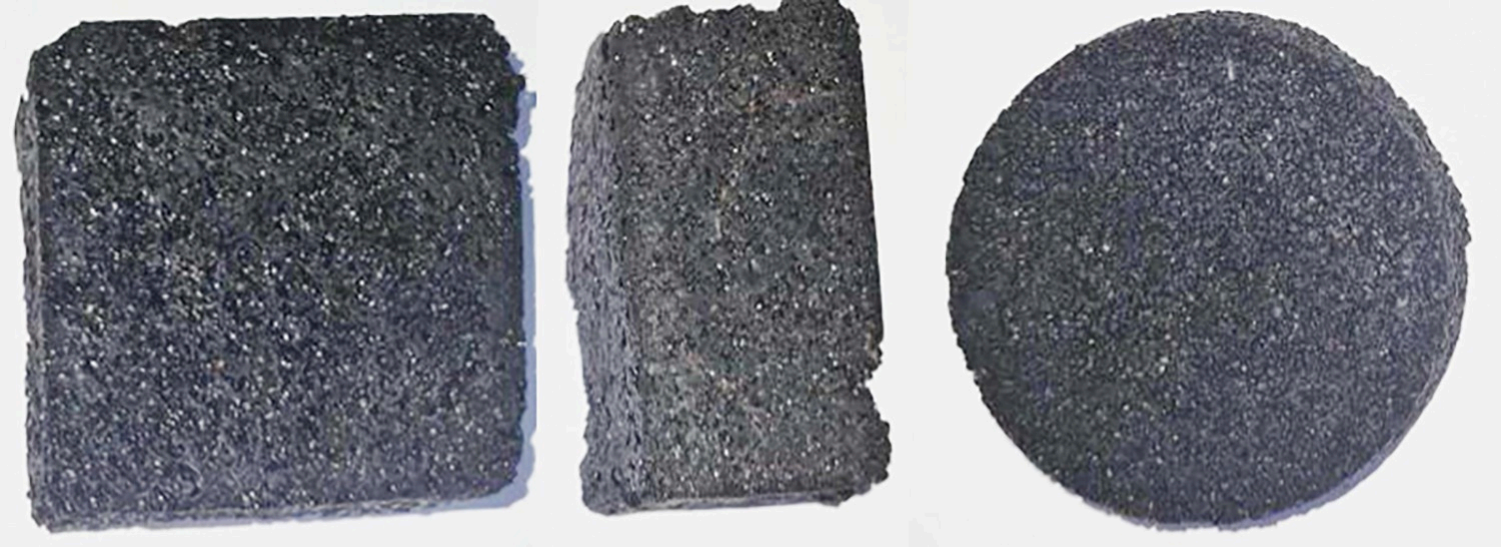
Photograph of composite with curly sorrel root (99CS).

Oil composites are characterised by a solid and porous structure and a dark brown colour (regardless of the expansion of the additive). In Fig 1, the use of a composite with curly sorrel roots is shown. In the abbreviations used, the number indicates the preparation of the composite according to Table 1, and the letter indicates the type of natural additive used: CS–curly sorrel and H–hops.

**Fig 2:**
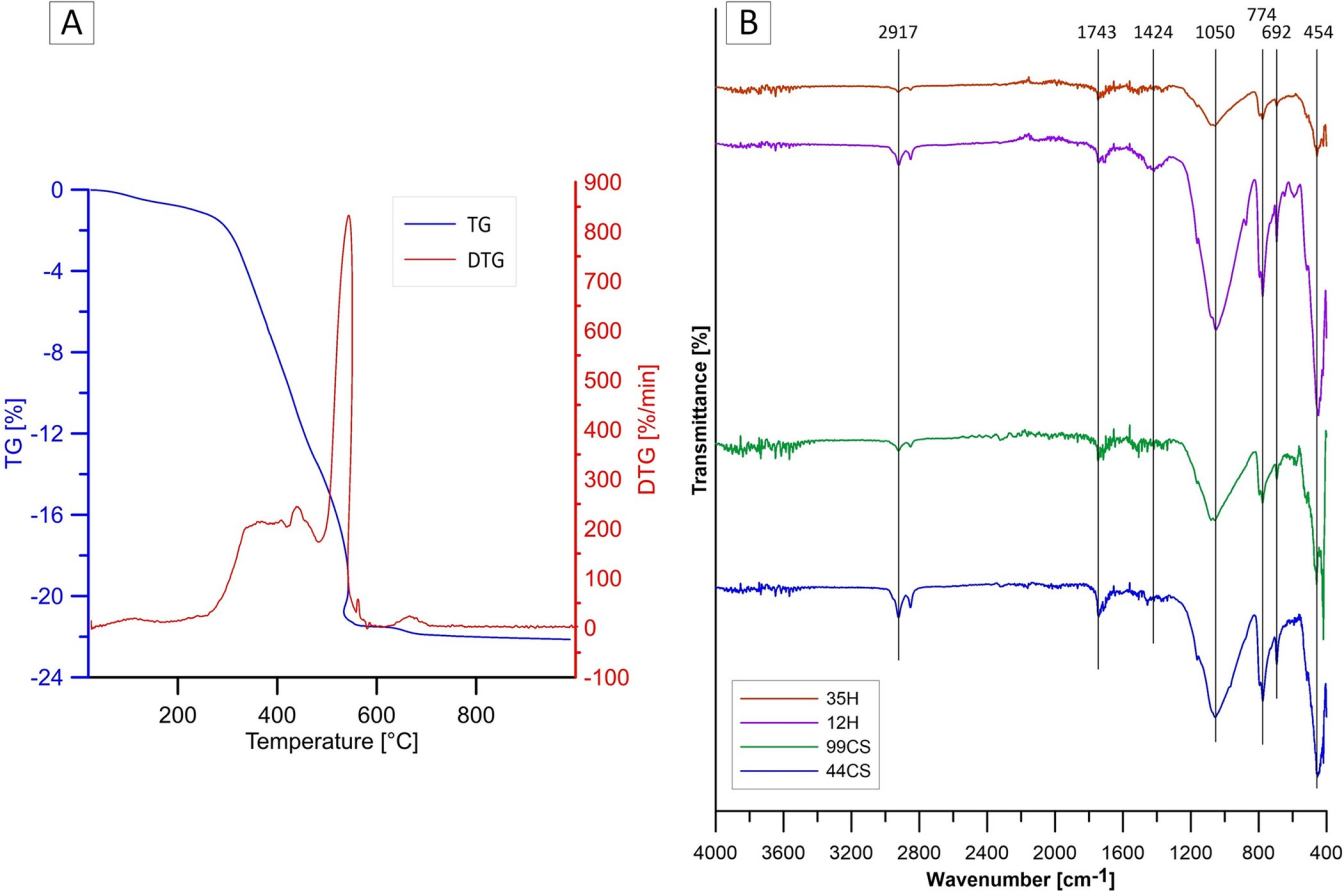
(A) Thermogravimetric/differential thermal analysis of composite 99CS; (B) FT-IR spectra of composites 12H, 35H, 44CS and 99CS.

The results of the thermogravimetric/differential thermal analysis and a photograph of the 99CS composite are shown in Fig 2A. The initial weight loss of the composite is due to the loss of water absorbed by this material from the environment. In the temperature range of 250–600°C, the highest weight loss is observed due to the decomposition of the sample components. Degradation of polyesters occurs in the temperature range of 200–350°C. Around 600°C, the oxidation reactions of double bonds in long chains of fatty acids take place. Above 600°C, the mass of the sample stabilises [25–27].

The FT-IR spectra of selected composites enriched with hops and curly sorrel root is shown in Fig 2B. The materials differ in both composition and annealing parameters. Signals at 2917 and about 2840 cm^-1^ were symmetric and asymmetric stretching vibrations of the CH_2_ group. The bands with a wavenumber of about 1743 cm^-1^ correspond to C = O stretching vibrations of the ester group, 1424 cm^-1^ to C = C stretching vibrations of the alkenyl group, and 1070–1050 cm^-1^ from C-O stretching vibrations. The bands for wavenumber 796–774 cm^-1^ are attributed to C = H deformation vibrations. Bending vibrations of C-H bonds of single-substituted aromatic compounds are observed in the form of bands with a wavenumber of 692 cm^-1^, while vibrations associated with torsional motion in organic molecules are around 454 cm^-1^ [28–31].

**Fig 3:**
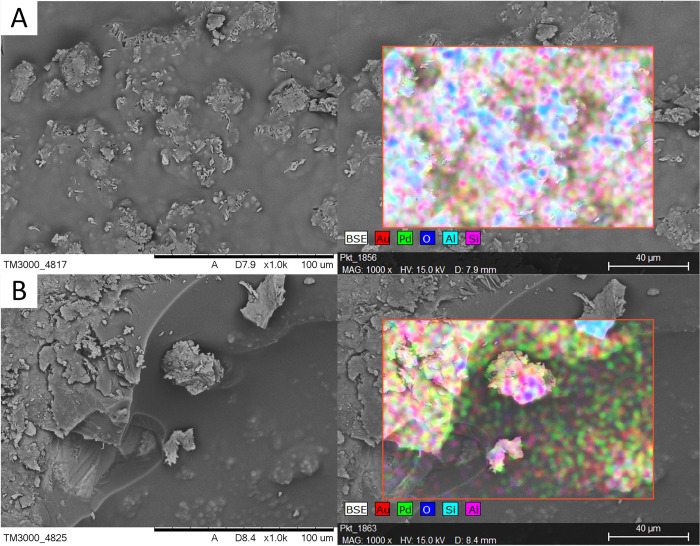
SEM micrograph and EDS analysis of composites (A) 12H and (B) 99CS.

Fig 3 shows an SEM micrograph and EDS analysis of composites 12H and 99CS. On the surface of the samples, the elements included in the composites’ compounds were identified as aluminum and silicon from sand, sulphur from sulfuric acid, carbon from WCO, and also palladium and gold from sputtering of the samples.

**Fig 4:**
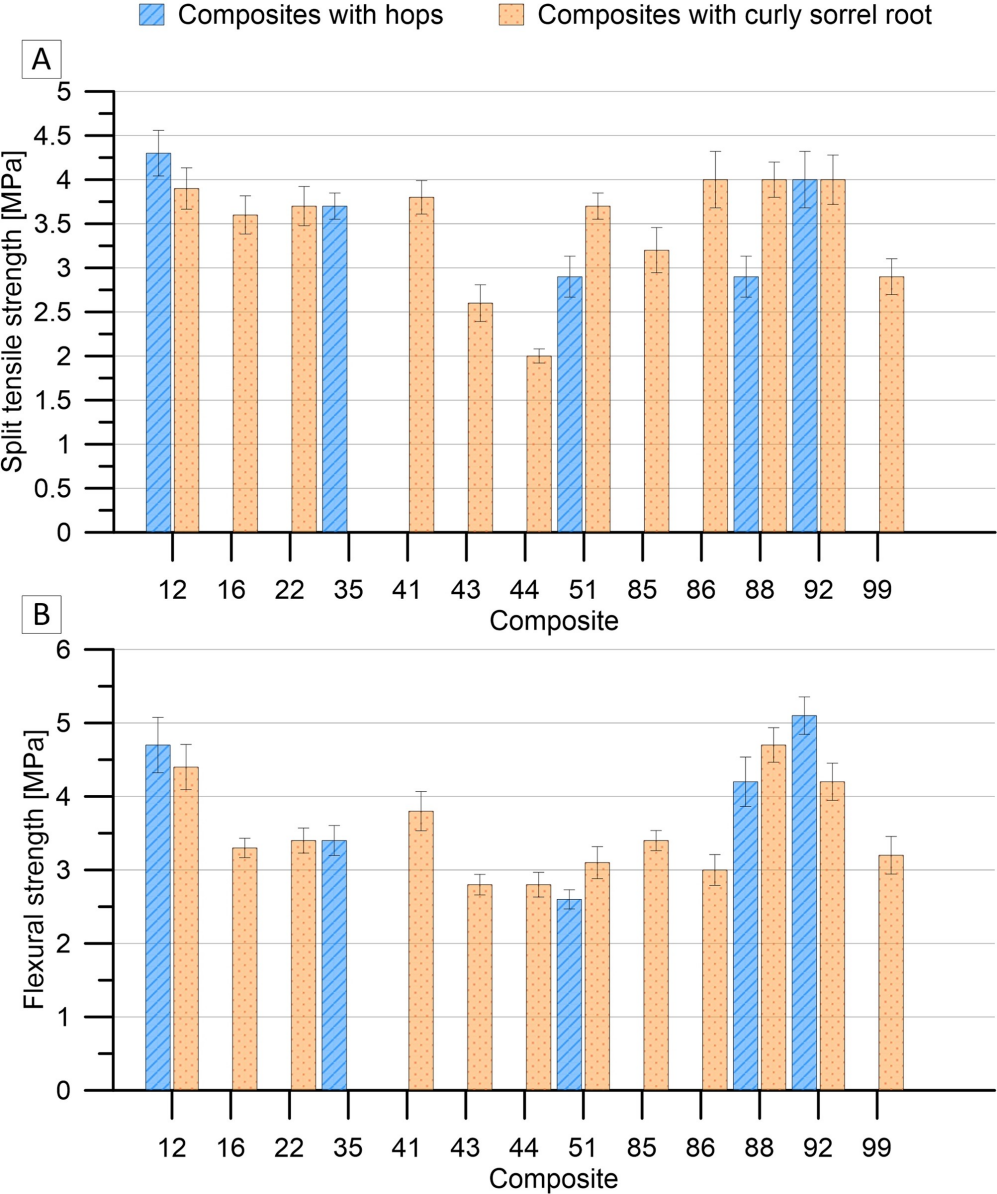
Split tensile (A) and flexural strengths (B) of oil composites.

The lowest split tensile strength at break, 2.9 MPa, was recorded for composites 51H, 88H, and 99CS, with a catalyst-to-oil mass ratio of 0.24. These composites were obtained at temperatures of 200°C and 210°C, with the addition of a natural ingredient of at least 4%. In turn, the highest tensile strength (4.3 MPa) was achieved in the case of the 12H composite, containing 1% hop and heated at 190°C for 20 hours (Fig 4A). It is worth noting that the strength properties of these composites can be compared with the properties of paving stones containing basalt minerals in an amount of 25–35% [32].

Regarding flexural strength, similar relationships were observed. Composite 92H, which was characterised by the highest bending strength (5.1 MPa), was obtained by heating at 210°C for 19 hours. This composite contained 1% hops and the mass ratio of acid to catalysed oil was 0.24 (Fig 4B). The oil blocks with the highest mechanical strength were distinguished by the highest mass ratio of acid catalyst to catalysed oil, which contributed to faster polymerisation and hardening of the samples.

**Fig 5:**
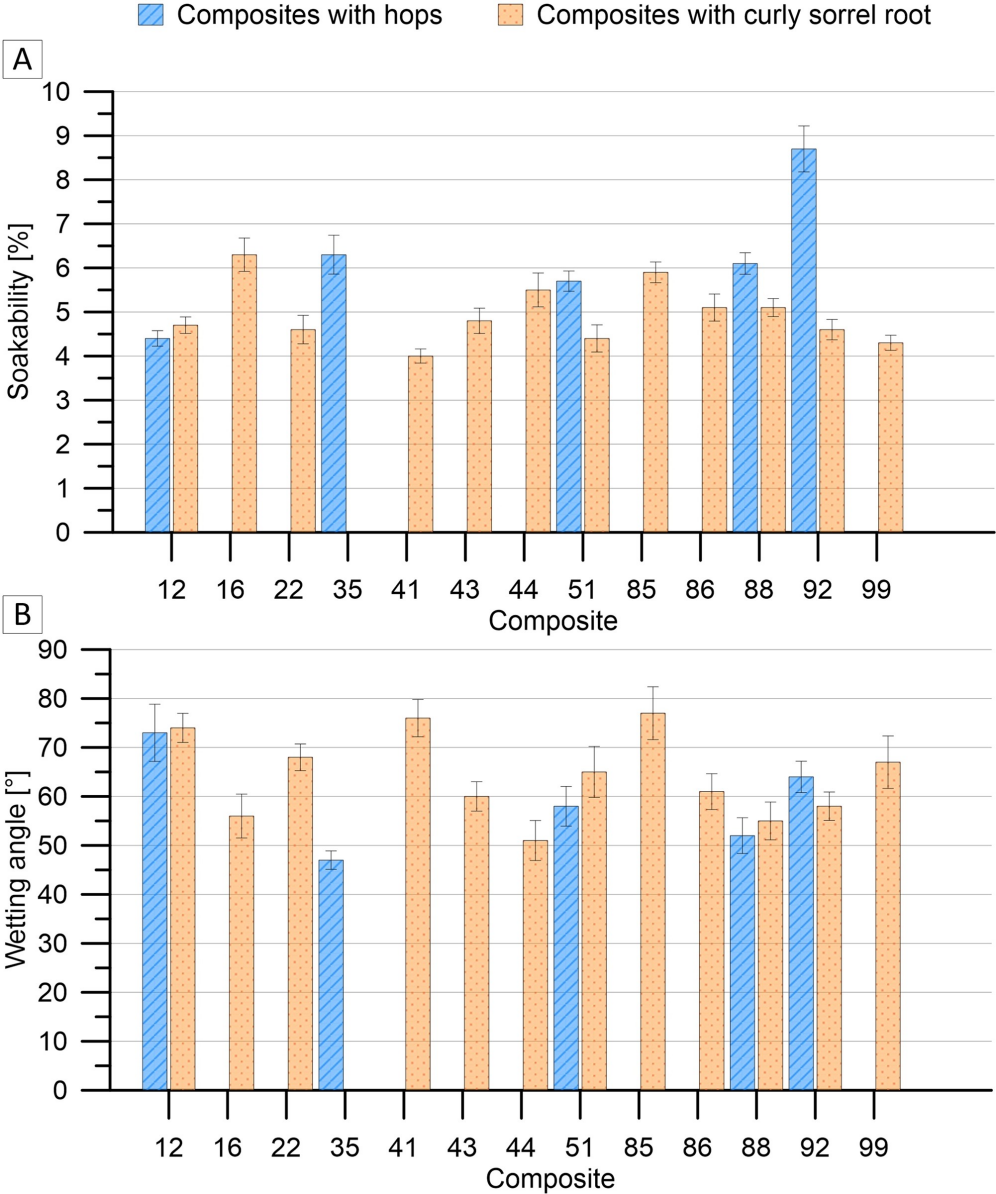
Soakability (A) and wetting angle (B) of oil composites.

The composites with hops had a greater variation in soakability and wetting angle than the composites with curly sorrel root (Fig 5A and 5B). The 12H composite annealed at 190°C for 20 hours containing 1% hops and characterised by an acid/catalysed oil mass ratio of 0.24 showed the lowest absorbability of over 4% but, at the same time, the highest surface wetting angle with water (74%). The absorbability of curly sorrel root composites was comparable, ranging from 4.3 to 5.1%.

### Antibacterial properties of oil composites

**Fig 6:**
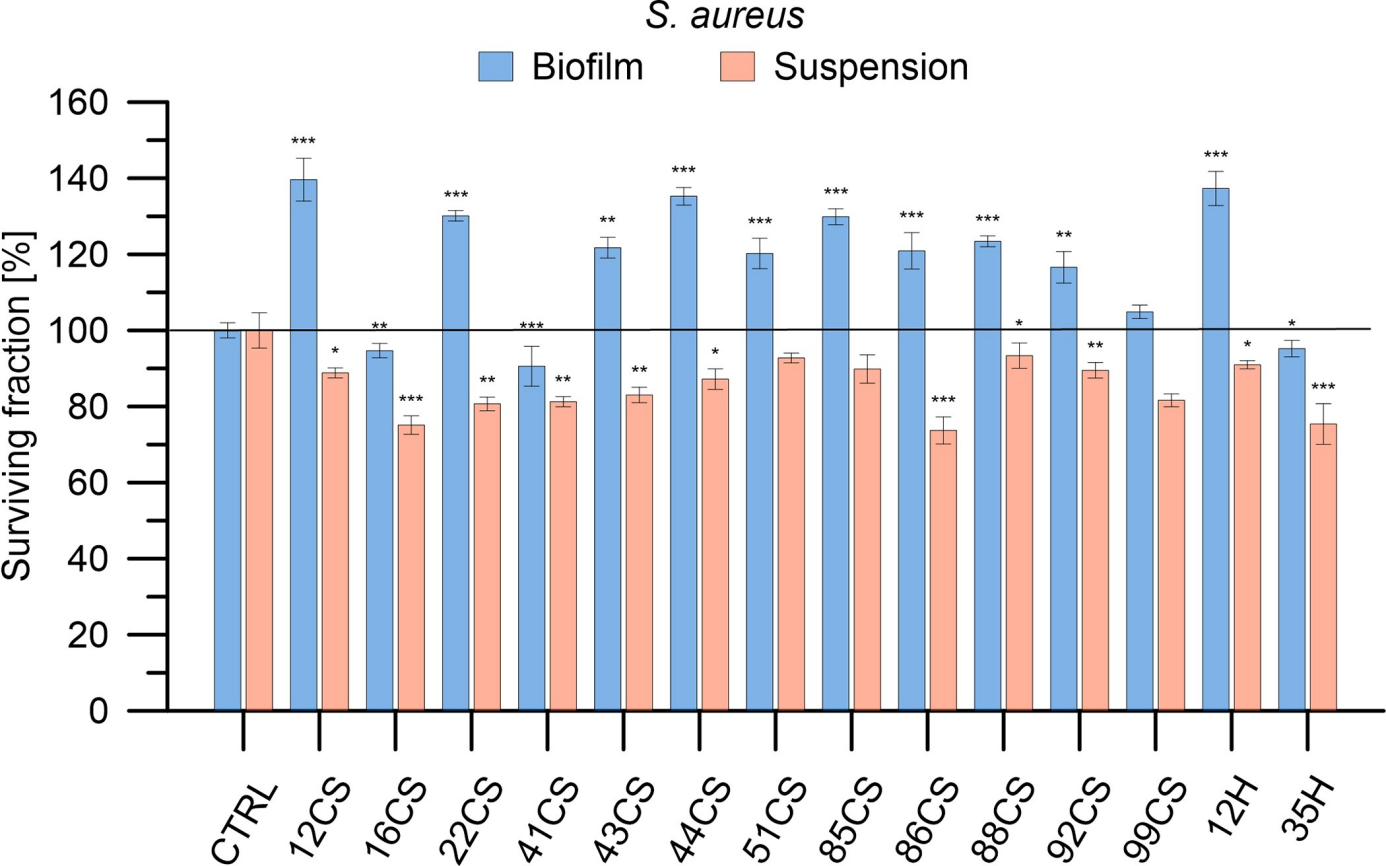
Determination of antibacterial activity of investigated composite materials against *S*. *aureus* in planktonic cultures and biofilm. *S*. *aureus* cells were exposed to each material in concentrations of 100 mg/mL for 24 h. Bacterial viability is expressed as a percent of the viability of the control (non-treated bacteria). Data are presented as mean ± SD The asterisks denote p-values < *0.05, **0.01, and ***0.001 compared to the control.

**Fig 7:**
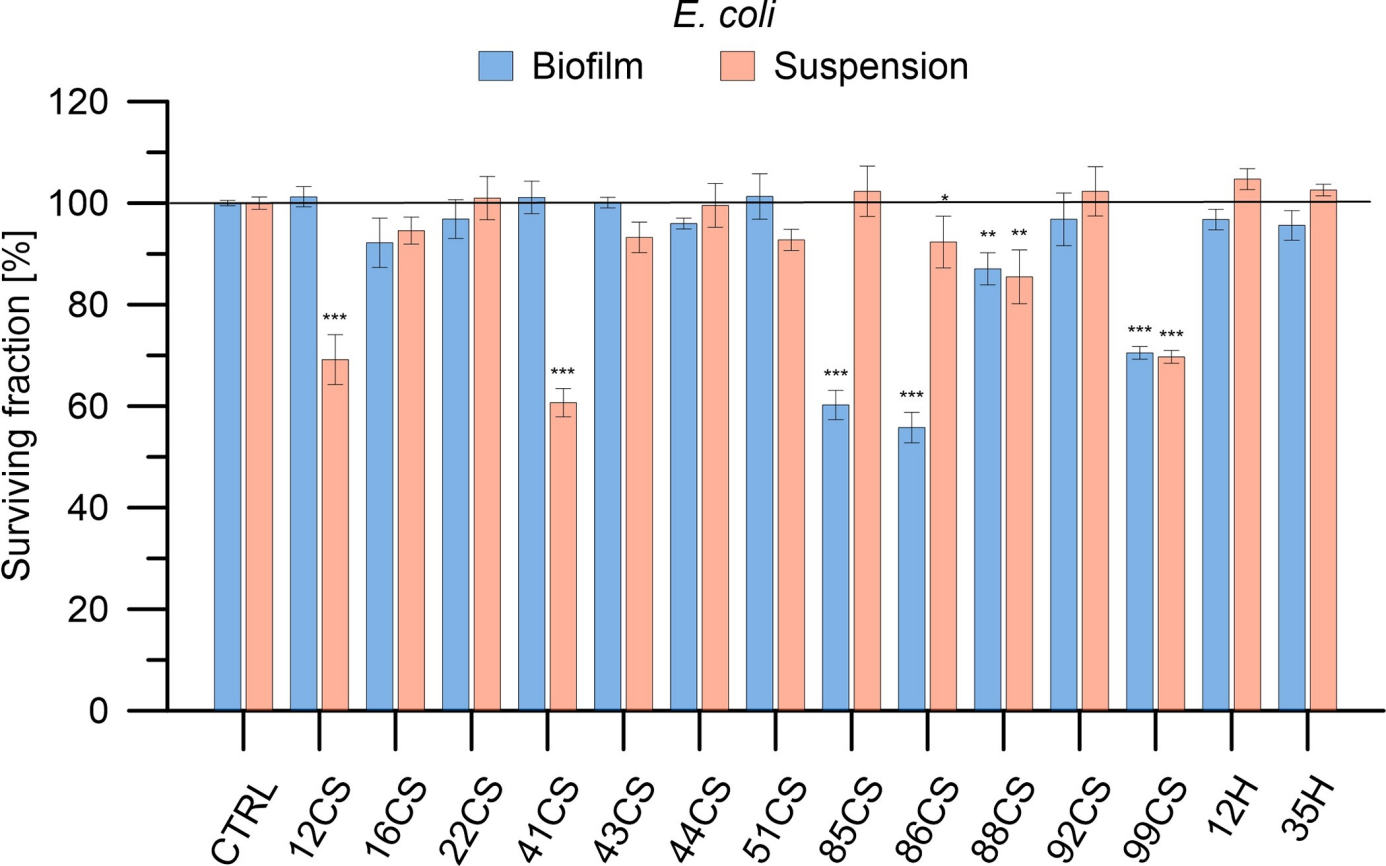
Determination of antibacterial activity of the investigated composite materials against *E*. *coli* in planktonic culture and biofilm. *E*.*coli* cells were exposed to each material in concentrations of 100 mg/ml for 24 h. Bacterial viability is expressed as a percent of the viability of the control (non-treated bacteria). Data are presented as mean ± SD. The asterisks denote p-values < *0.05, **0.01, and ***0.001 compared to the control.

**Fig 8:**
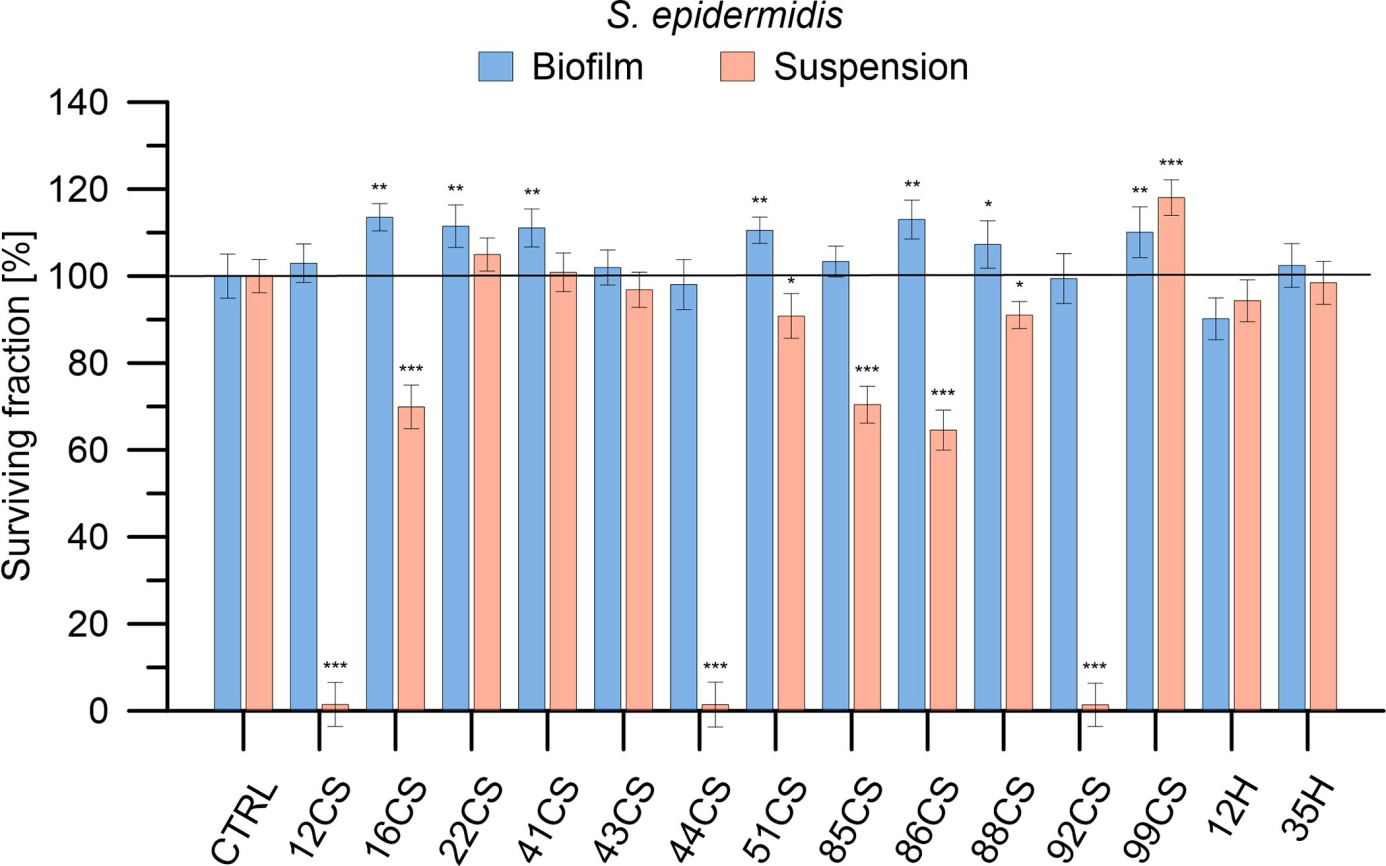
Determination of antibacterial activity of the investigated composite materials against *S*. *epidermidis* in planktonic culture and biofilm. *S*. *epidermidis* cells were exposed to each material in concentrations of 100 mg/mL for 24 h. Bacterial viability is expressed as a percent of the viability of the control (non-treated bacteria). Data are presented as mean ± SD. The asterisks denote p-values < *0.05, **0.01, and ***0.001 compared to the control.

**Fig 9:**
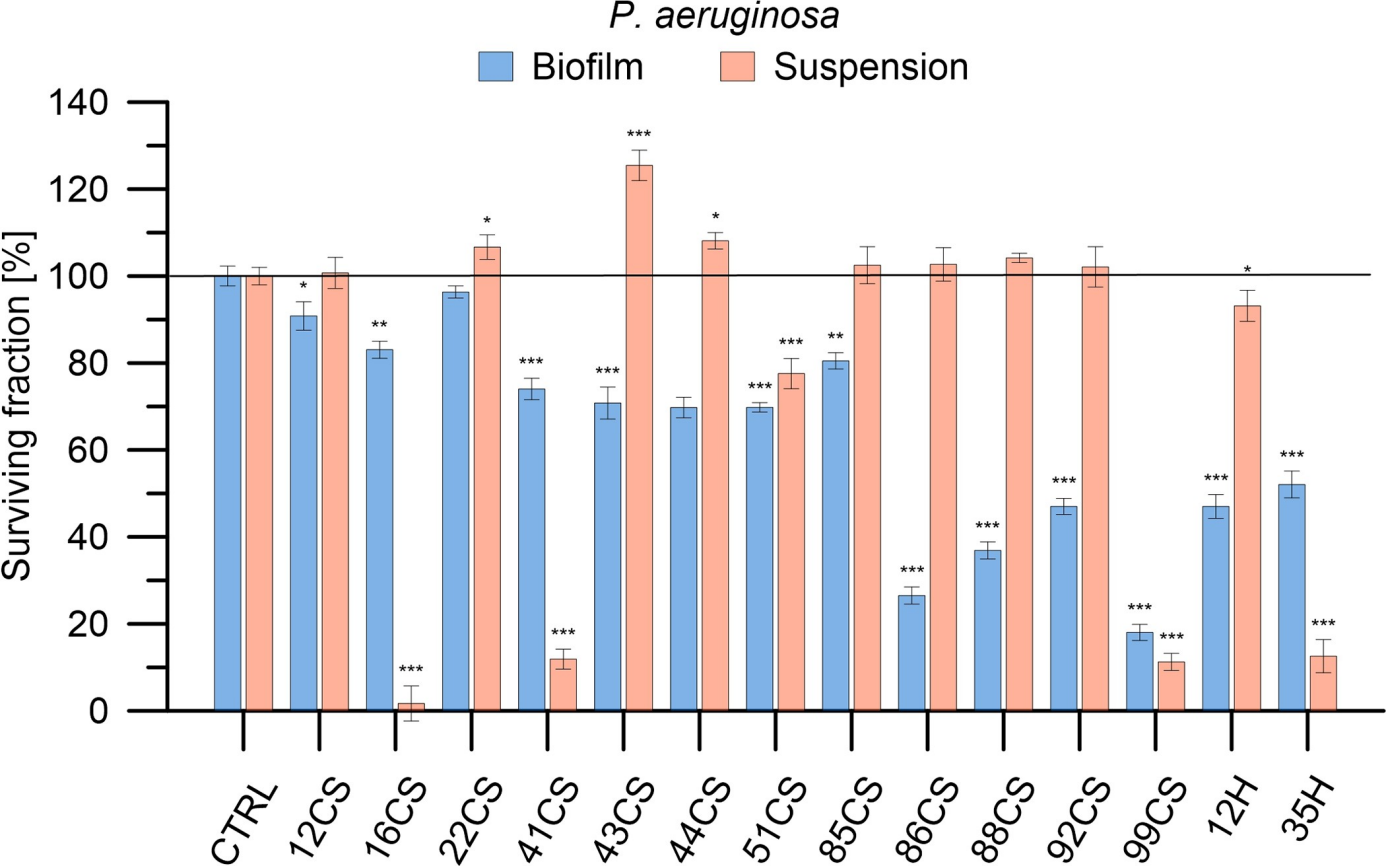
Determination of antibacterial activity of the investigated composite materials against *P*. *aeruginosa* in planktonic culture and biofilm. *P*. *aeruginosa* cells were exposed to each material in concentrations of 100 mg/mL for 24 h. Bacterial viability is expressed as a percent of the viability of the control (non-treated bacteria). Data are presented as mean ± SD. The asterisks denote p-values < *0.05, **0.01, and ***0.001 compared to the control.

**Fig 10:**
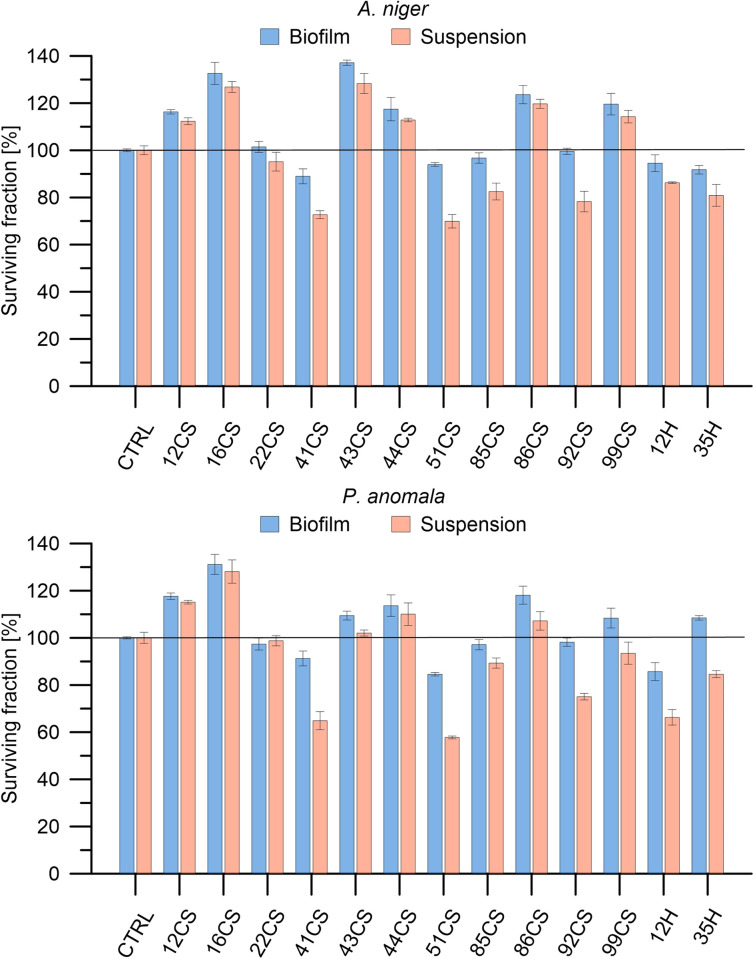
Determination of the antifungal activity of investigated composite materials modified with hops (H) and curly sorrel (CS) against *P*. *anomala* and *A*. *niger* in planktonic culture and biofilm was performed. In each case, fungi were exposed to each material at a concentration of 100 mg/mL for 24 h. Fungal viability was expressed as a percentage of viability compared to the control (non-treated fungi). Data are presented as means ± SD.

**Fig 11:**
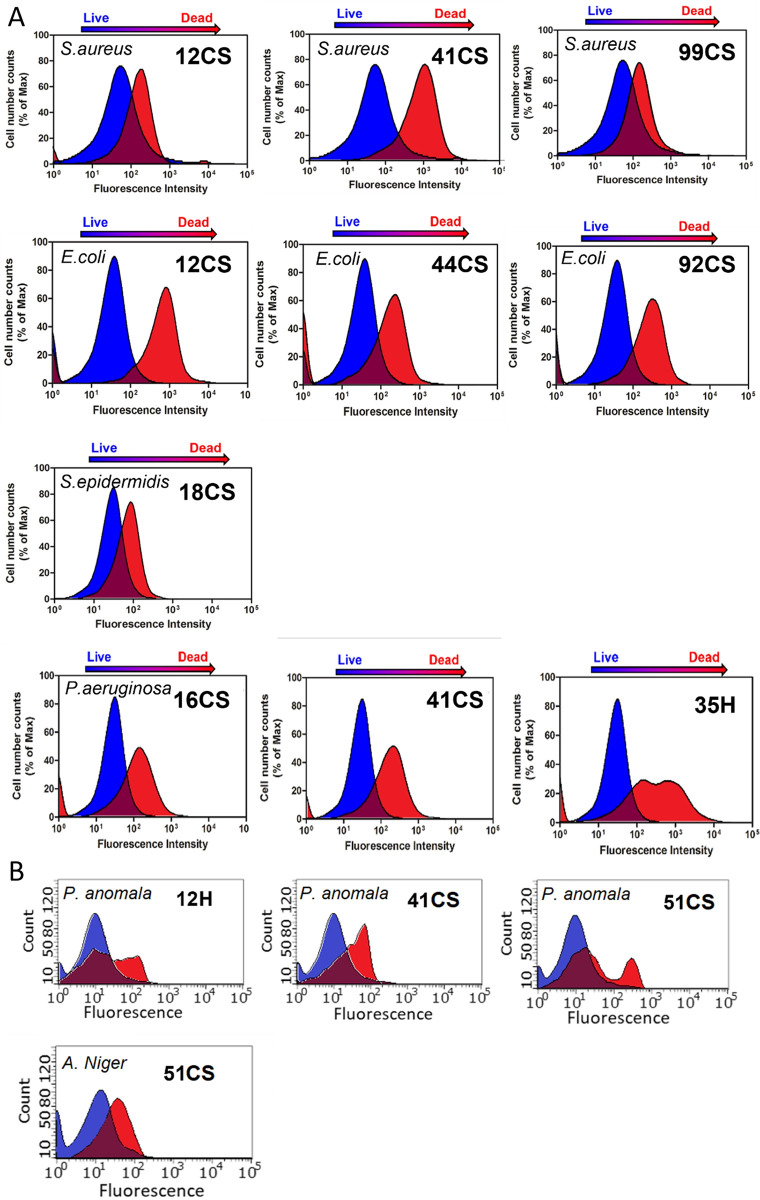
Flow cytometry analysis of (A) bacteria and (B) fungi after 24 hour treatment with investigated composite materials. For this purpose, microorganisms were stained with propidium iodide (10 ug/mL) after treatment and the red fluorescence of dead cells was detected.

The antimicrobial efficacy of 12 composite materials enhanced with curly sorrel and 2 composites enhanced with hop was assessed against Gram-positive bacteria (*S*. *aureus*, *S*. *epidermidis*), Gram-negative bacteria (*E*. *coli*, *P*. *aeruginosa*), and fungi (*A*. *niger*, *P*. *anomala*) microorganisms. The applied abbreviations indicate the percentage content of the modifier, with the number being the % and the letters CS representing curly sorrel and H representing hop. A concentration of 100 mg/mL was employed for each substance. The bacteria and fungi were cultivated in both planktonic and biofilm states. Microorganisms were exposed to the materials for a duration of 24 hours, after which the survival rate of the bacteria or fungi was evaluated. Figs 6–11 demonstrate the efficacy of the materials in inhibiting the growth of all strains tested. In addition, acknowledging the fact that microorganisms in biofilms exhibit a resistance to antimicrobial drugs that is 10–1000 times higher than planktonic bacteria and fungi, we conducted a study to examine the effectiveness of composite materials against bacterial and fungal biofilms. Biofilms were formed by incubating the microorganisms in 12 flat bottom microplates that were covered with a thin coating of agar for 24 hours. Following the formation of the biofilms, they were exposed to composite materials at a concentration of 100 mg/mL for 24 hours (seen in the bottom panels of Figs 6–11). The effectiveness of antimicrobial medicines may be quantified using minimum inhibitory concentrations (MICs). Therefore, the MIC values were established for the most active materials, as shown in Table 2.

**Table 2:** MIC values determined for the most active materials against all tested bacteria strains.

Strain	Material	MIC (mg/mL)
*S*. *epidermidis*	12CS	69.54
*S*. *epidermidis *	44CS	81.84
*S*. *epidermidis *	92CS	82.08
*P*. *aeruginosa *	16CS	46.03
*P*. *aeruginosa *	41CS	52.05
*P*. *aeruginosa *	99CS	49.55
*P*. *aeruginosa *	35H	60.83

It is crucial to acknowledge that the precise antimicrobial effectiveness of waste cooking oil composites may vary based on the composition and intended use. Conducting experimental investigations and testing is essential in order to accurately assess the antimicrobial effectiveness of a certain composite system that is derived from waste cooking oil. According to the results provided, it can be inferred that the higher proportion of modifier content (both CS and H) does not enhance the antimicrobial effectiveness of the material. For example, the presence of 16CS significantly decreased bacterial viability by around 70%, but 22CS had the opposite effect and increased bacterial activity. Therefore, it indicates that the antibacterial activity is strongly connected to the other physical and chemical characteristics of the composite material and arises from the combined impact of the base material and the modifier.

All materials treated with curly sorrel induced the inactivation of *S*. *aureus* bacteria. Out of all the options, 86CS had the highest level of activity, resulting in a reduction in viability by as much as 27%. The 16CS substance resulted in a reduction in bacterial activity by around 20–25%. Additional substances, including 22CS, 41CS, 43CS, and 99CS, resulted in a reduction of bacterial viability by around 15–20%. Regarding the products containing the second modification, hop, both exhibited antibacterial activity against *E*. *coli*. 35H exhibited the highest level of activity, resulting in a 25% reduction in bacterial viability. The presence of additional active compounds, including 12H, resulted in a reduction in viability of around 10%, which may result from specific reactions of *S*. *aureus* consisting in extending the lag phase and prolonging the generation time under the influence of hop extracts in order to survive among the hop components [33].

It is noteworthy that both sets of materials had lower activity against bacteria that form biofilms, except for the 41CS material. The vitality of bacteria cultivated in suspension was comparable to that of bacteria cultured in biofilm, with both groups exhibiting around 80% viability.

The susceptibility of Gram-negative and Gram-positive bacteria to composite materials based on waste cooking oil can vary due to inherent differences in their cell wall structure and composition. That is why the antimicrobial activity of materials was also tested against Gram-negative species.

For *E*. *coli*, 8 of the materials tested showed antibacterial properties. However, only three compound (12CS, 41CS, and 99CS) showed significant activity resulting in 60–70% bacterial inactivation. The others (16CS, 43CS, 51CS, 85CS, and 88CS) caused mortality of around 10% of the bacterial population. In the case of materials with hop, their activity was insignificant. In the case of bacteria growing as a biofilm with 3 materials (85CS, 86CS, and 99CS), a decrease in viability of up to 60% was recorded.

In the case of *S*. *epidermidis* generated in a suspension culture of nine materials, an antibacterial effect was demonstrated. In materials 12CS, 44CS, and 92CS, the effect of the reduction was significant, even to 99%. In the case of biofilm, no material exhibited biological activity. For the biofilm, a slight effect on the microbiological activity of the material 12H was observed. The biocidal nature of hops results from the hydrophobic nature of the compounds contained in hop extracts, thanks to which hop ingredients penetrate the bacterial cell wall, and then, as a result of the interaction of these ingredients with the inner membrane, the cellular structure is damaged, which inhibits the active transport of sugars and amino acids [34]. This fact results in higher antibacterial activity of hop extracts against Gram-positive bacteria [35]. For materials 12CS, 44CS, and 92CS, it was possible to determine MIC values of 69.54, 81.84, and 82.03 mg/mL, respectively.

The strain most susceptible to the tested materials is *P*. *aeruginosa*. Against bacteria growing in suspension, as many as 3 materials caused inactivation of bacteria around 90% (16CS (99%) > 99CS (88%) > 41CS (88%)). The most active material was 16CS (97% inactivation). Material with moderate activity also included 51CS. The antibacterial effect observed for them was 23% inactivation. The hop group materials were also most active against *P*. *aeruginosa*. The 35H material caused almost complete inactivation of the bacteria (an inactivation level of 88%).

Interestingly, the tested materials appeared to be active against *P*. *aeruginosa* biofilm. Most curly sorel-modified materials caused a significant decrease in biofilm viability. The effect varied, and the recorded decrease in viability was in the range of 20–80%. The activity of the materials in descending order can be rearranged as follows: 99CS > 86CS > 88CS > 92CS > 12H > 35H > 51CS > 44CS > 43CS > 41CS > 16CS > 85CS > 12CS). For 4 compounds, MIC values were obtained: 16CS– 46.03 mg/mL, 41CS– 52.05 mg/mL, 99CS– 49.55 mg/mL, 35H – 60.83 mg/mL.

### Antifungal properties of oil composites

The tested composites subjected to antifungal tests showed that only some of the composites made with curly sorrel induced the inactivation of the fungi *A*. *niger* (22CS, 41CS, 51CS, 85CS, 92CS) and *P*. *anomala* (22CS, 41CS, 51CS, 85CS, 92CS, 99CS) (Fig 10). However, in the case of composites made with hops, both were characterised by inactivation of the tested fungi in suspension, while in the biofilm only in relation to *A*. *niger*. Among the tested composites, the 41CS and 51CS composites had the highest level of activity in suspension. Reductions in fungal viability were observed in the 41CS composite by 35% and 27%, and in the 51CS composite by 42% and 30% for *P*. *anomala* and *A*. *niger*, respectively. In the case of composites containing hops, the 12H composite had the highest fungal inactivation for *P*. *anomala*. A reduction in the viability of this fungus by approximately 34% was observed, and, in the case of *A*. *niger*, by approximately 14%. The 35H composite reduced the activity by 15% and 19% for *P*. *anomala* and *A*. *niger*, respectively. It is worth noting that the activity of the tested composites differed in relation to biofilm-forming and suspension-forming fungi. A similar trend is observed as in the case of tests with bacteria, i.e., in the case of tests conducted in suspension, the composites showed greater activity than in the case of fungi forming biofilms.

To conduct further antibacterial activity experiments, we examined the survival of bacteria after treatment with certain composite materials using flow cytometry (Fig 11). Therefore, we chose the most representative materials based on their highest level of inactivation in each microbe investigated. Following the incubation period with the substance, the bacteria were collected and treated with propidium iodide in order to see the dead cells.

Propidium iodide is unable to permeate viable microorganisms with intact cell membranes, but it may penetrate bacteria and fungi with defective or broken membranes. It inserts itself into the DNA, causing a red fluorescence signal to be produced. Therefore, in this experiment, the fluorescence generated by bacteria and fungi labelled with propidium iodide, after treatment with certain composite materials, may be recorded and studied using flow cytometry. Typically, the brightness of fluorescence in the red channel is indicative of propidium iodide staining, which signifies the presence of dead or permeabilised microorganisms. The flow cytometry plot reveals distinct populations, including living cells (PI-negative) and dead cells (PI-positive), as shown by the findings. More red-shifted fluorescence indicates a higher proportion of dead cells. Compound 12CS was identified as the most poisonous substance for the bacterium *E*. *coli* and 35H against *P*. *aeruginosa*. However, in the case of tests conducted against fungi, the 51CS composite showed the highest toxicity to *P*. *anomala*.

**Fig 12:**
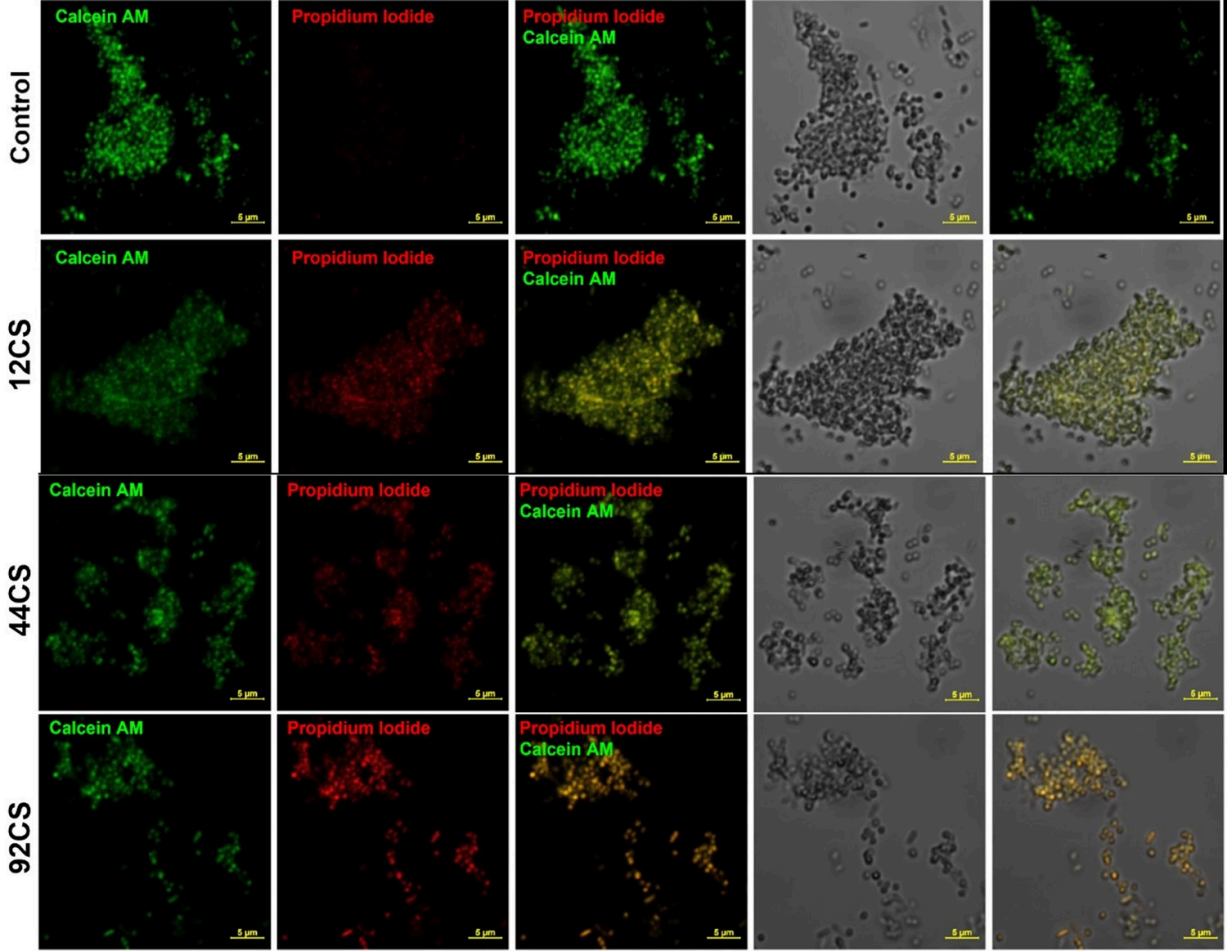
Laser scanning confocal microscopy images of the non-treated (upper panel) and selected composite material-treated (bottom panels) S. epidermidis stained with Calcein AM and propidium iodide with bright-field (T-PMT) contrast.

**Fig 13:**
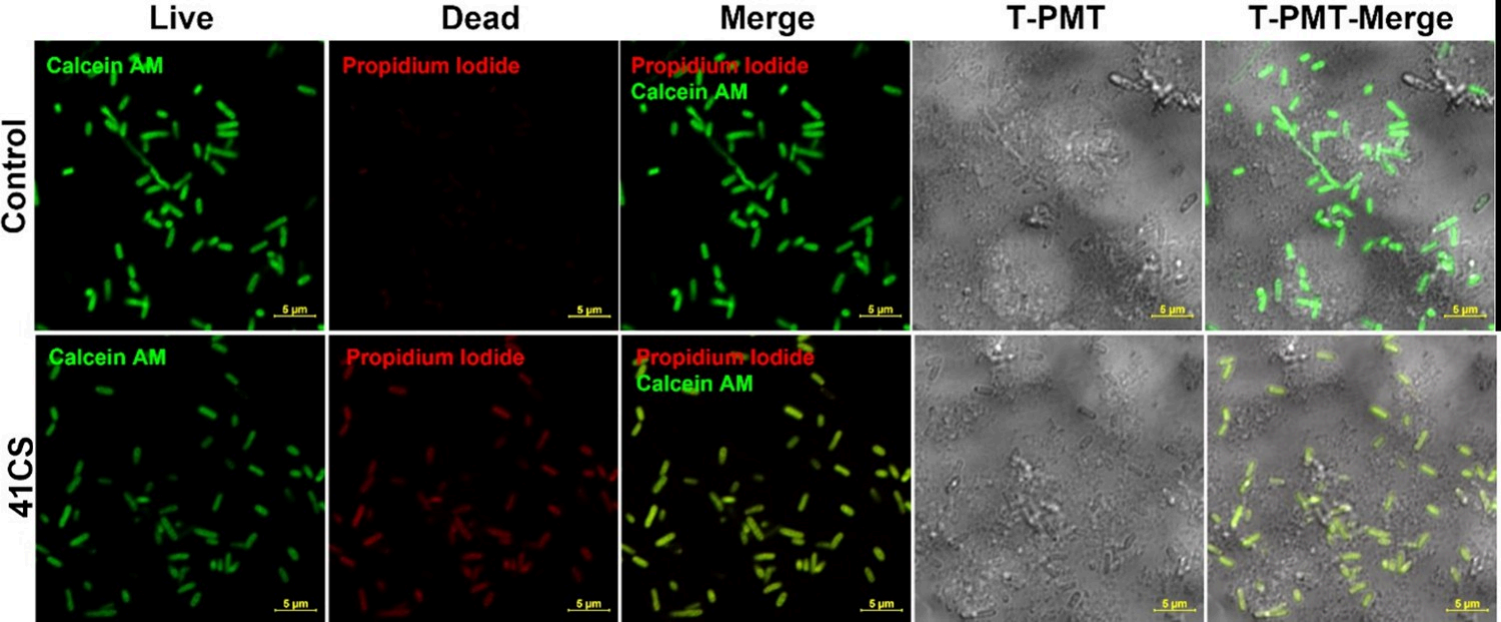
Laser scanning confocal microscopy images of the non-treated (upper panel) and selected composite material-treated (bottom panels) *P*. *aeruginosa* stained with Calcein AM and propidium iodide with bright-field (T-PMT) contrast.

**Fig 14:**
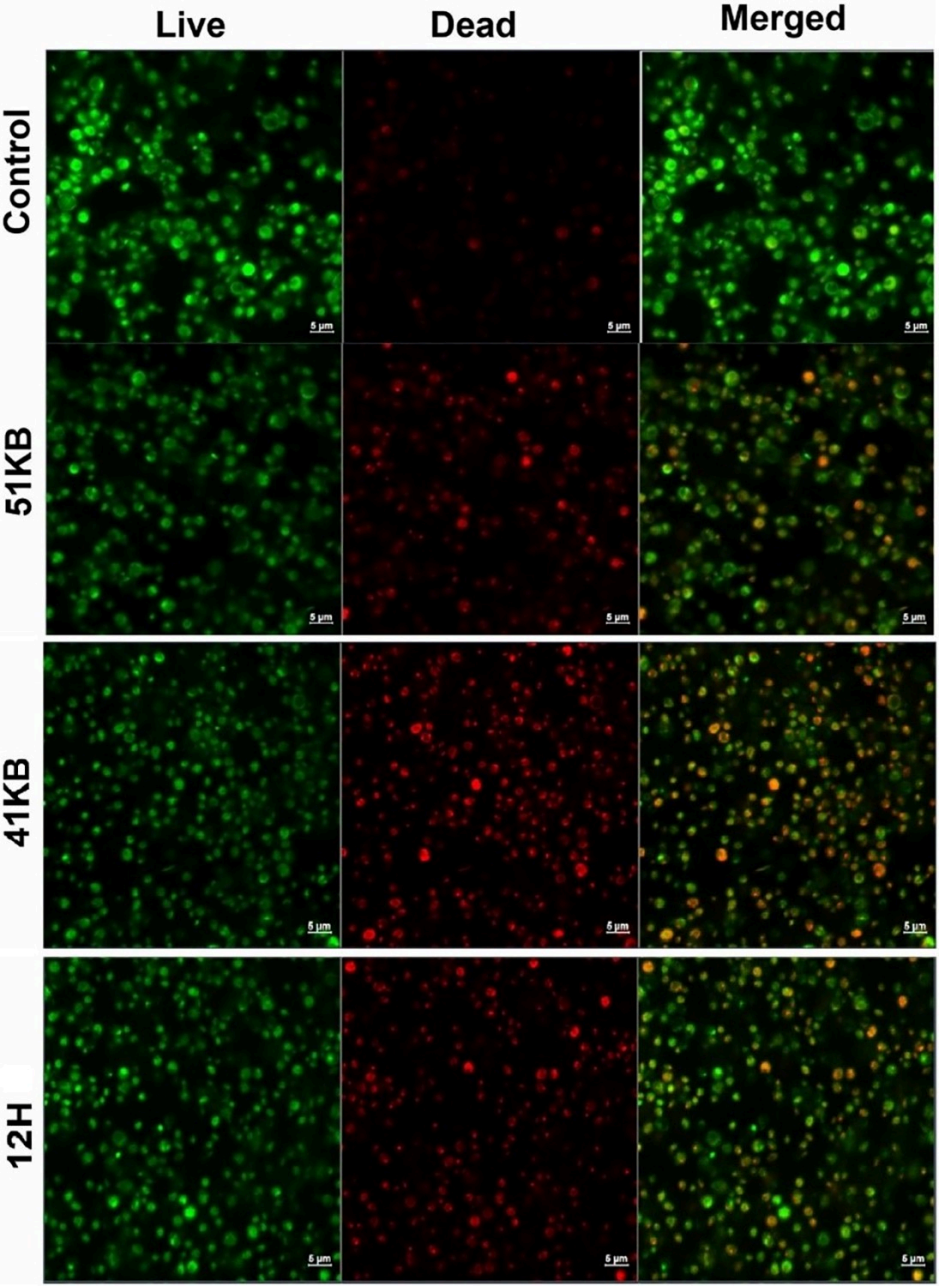
Laser scanning confocal microscopy images of the non-treated (upper panel) and selected composite material-treated (bottom panels) *P*. *anomala* stained with Calcein AM and propidium iodide.

**Fig 15:**
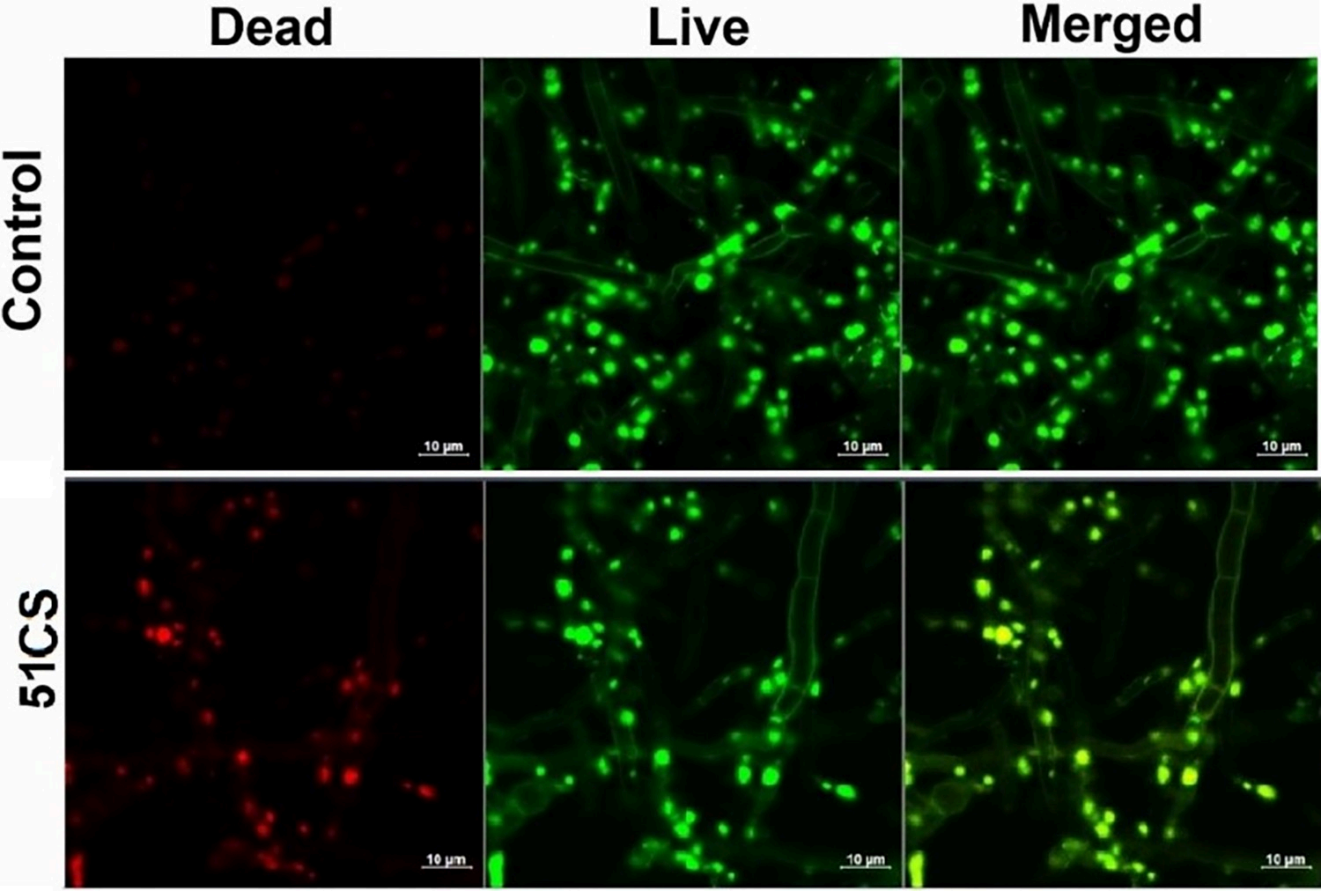
Laser scanning confocal microscopy images of the non-treated (upper panel) and selected composite material-treated (bottom panels) *A*. *niger* stained with Calcein AM and propidium iodide.

In order to further illustrate the extent of the damages caused and the variations in bacterial and fungal death processes, we conducted confocal imaging after the application of certain composites. Figs 12–15 show the typical confocal pictures of the bacteria and fungi that are most responsive to therapy, namely *P*. *aeruginosa*, *S*. *epidermidis*, *A*. *niger*, *and P*. *anomala*. The vitality of the bacteria and fungi was assessed in these investigations by staining them with Calcein AM and propidium iodide (PI) after treatment with certain composite materials. Calcein AM is a fluorescent dye with a green colour that is often used for labelling cells that are alive. Live cells, when stained with calcein, exhibit a vivid green glow when excited. This demonstrates the capacity of the cells to survive and function metabolically, as well as the presence of intact cell membranes, which enables the entry of calcein and the subsequent production of a fluorescence signal. In contrast, propidium iodide is a red fluorescent dye that specifically labels cells that are dead or have been made permeable. Dead bacteria and fungi, when stained with propidium iodide, will have a red glow. PI is unable to enter active cells that have intact cell membranes, but it can permeate the compromised or broken membranes of dead bacteria and fungi. It can be shown that the use of composites in therapy caused a breakdown of bacterial and fungal cells and may prevent the microorganisms from dividing without causing noticeable damage to the cell membrane. However, it is hypothesised that these bacteria and fungi were experiencing cell death or a decline in cellular functionality while still maintaining their cell walls intact.

**Fig 16:**
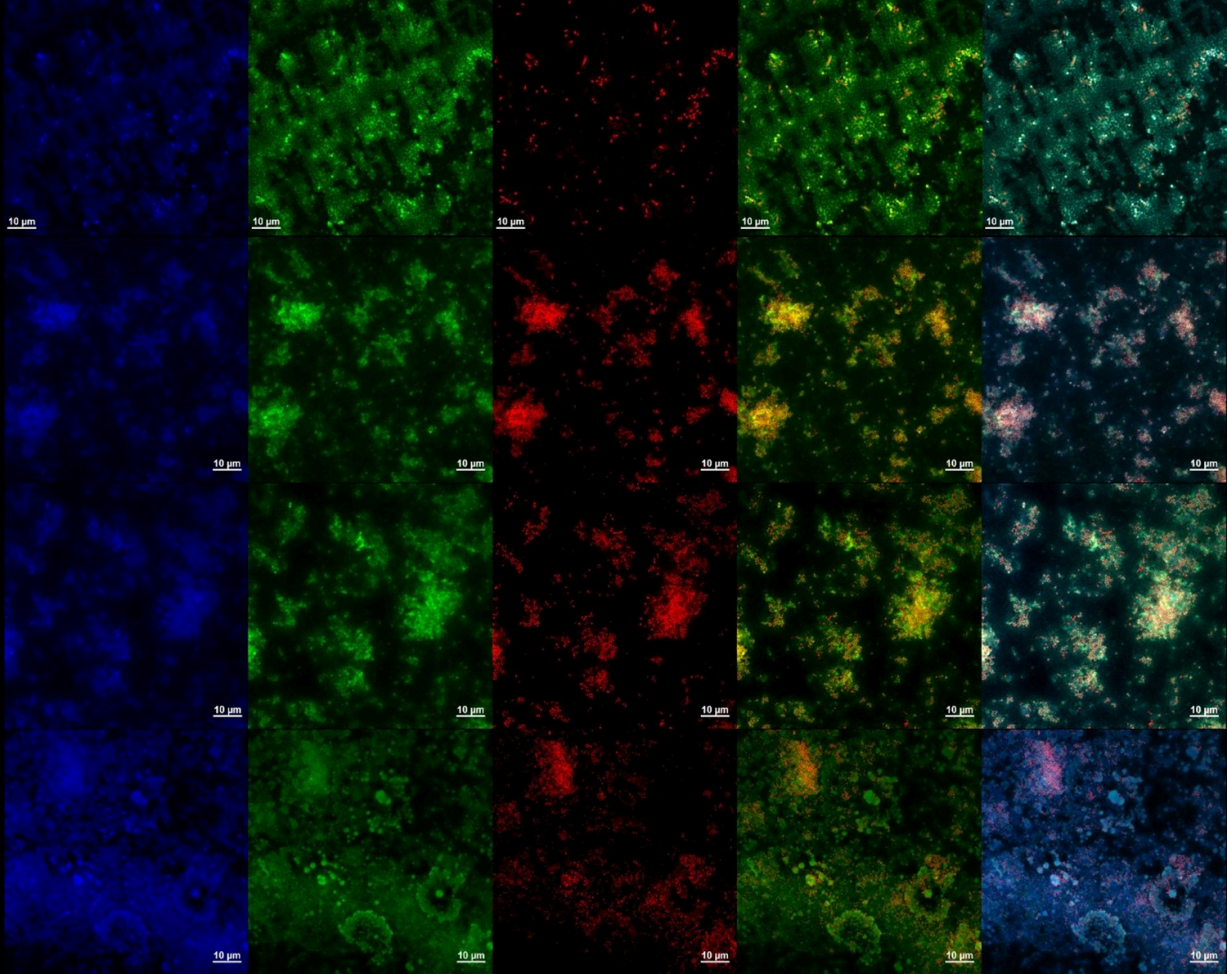
Laser scanning confocal microscopy images of the non-treated (upper panel) and selected composite material-treated (bottom panels) *S*. *aureus* biofilms stained with Hoechst 33342, Calcein AM, and propidium iodide.

To get a deeper understanding of the interactions between certain composite materials and biofilms, we used confocal microscopy to investigate the behaviour of *S*. *aureus* biofilms when incubated with the tested materials - 85CS, 86CS, and 99CS. Fig 16 displays sample photos of the biofilms after a 24-hour incubation period with these ingredients. For the subsequent procedure, the biofilms were treated with propidium iodide (PI) to stain dead cells, Calcein AM to stain living cells, and Hoechst dye to stain nucleic acids. Laser scanning confocal microscopy was used for imaging.

The presence of a biofilm may be recognised as island-like formations. The red fluorescence emitted by deceased bacteria seems to be located inside the biofilm, suggesting that the material’s activity may be ascribed to its surface contact and potential infiltration into the structure of the biofilm.

**Fig 17:**
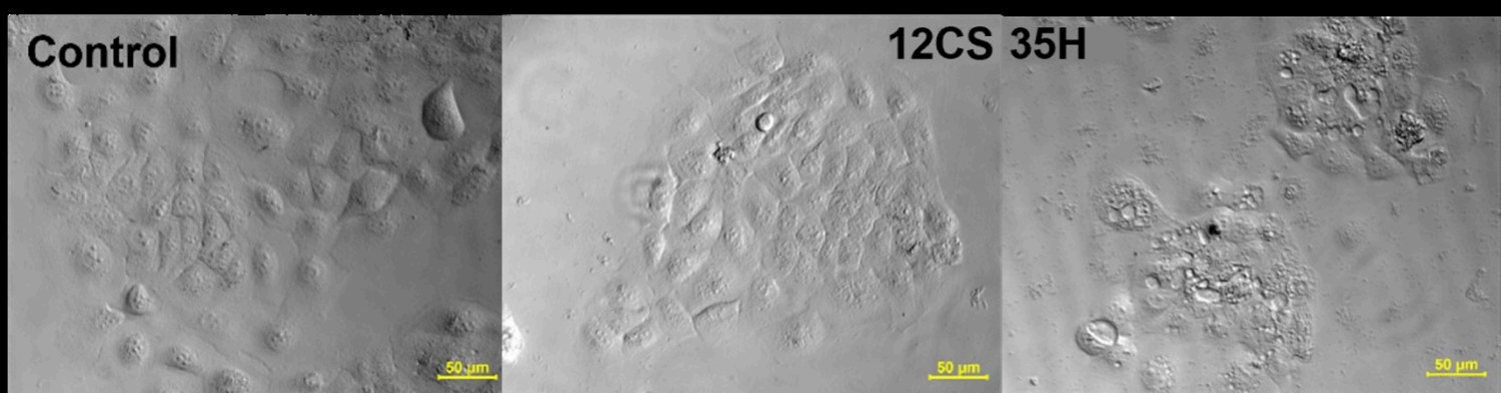
The morphology of HaCaT cells treated with selected composite materials visualised with bright-field microscopy.

The selectivity for microbial cells over host mammalian cells was demonstrated using the human keratinocytes cell line HaCaT. The viability of HaCaT cells and their morphology after treatment with composite materials after 24 hours of incubation is shown in Fig 17 (bright-field microscopy imaging).

According to the data obtained, it can be shown that the examined materials did not cause any substantial cell death or cell damage in human keratinocytes (HaCaT cells). The findings obtained from microscopic inspections suggest that HaCaT cells maintain their viability and continue to perform their regular biological functions even after exposure to certain compositions that exhibit antibacterial activity. This implies that the studied materials do not hinder the capacity of human cells to grow and multiply. Furthermore, the materials do not cause the production of hazardous compounds or elicit harmful reactions in human keratinocytes, such as cellular stress, oxidative harm, or DNA/RNA damage.

To summarise, the evaluated materials have been shown to be compatible with human keratinocytes, indicating that they are unlikely to produce any negative effects when in touch with human skin or other tissues rich in keratinocytes. Additionally, it implies that the substance might be considered safe for use or exposure in situations involving these cells, such as in the creation of skincare items or biomaterials designed for direct contact with the skin.

## Discussion

The physicochemical properties of composites are determined by the process parameters for obtaining these materials. The most important parameter is the heating time, the increase of which has a positive impact on the strength parameters, which is clearly visible in the case of bending strength. In the case of tensile strength, additional parameters such as the heating temperature and the mass ratio of sulfuric acid to WCO are important. It was also observed that an increase in the content of natural additive in the composite reduces the mechanical strength of the material. Ardila-Suárez et al. [36] presented the results of research on the influence of sulfuric acid on the reaction of forming a polymer compound. In a combinatorial experiment, the influence of temperature and catalyst concentration on the polyglycol polymerization process was investigated. By controlling process parameters, the properties of the products were modified, as is the case with composites based on waste cooking oil.

The antimicrobial properties of hops result from the presence of α-acids (humulones) and β-acids (lupulones) [37] At high temperature, α-acids (cohumulone, humulone and adhumulone) undergo isomerization to iso-α-acids, which have antibacterial activity, mainly against Gram-positive bacteria [38]. Moreover, approximately 10% of the dry weight of hops are equivalents of β-acids (colupulone, lupulone and adlupulone) with antimicrobial properties [34]. The antibacterial effect of hop ingredients consists in inhibiting the replication of microorganisms such as bacteria, fungi, protozoa, and parasites. A similar mechanism of action has been observed for some virulence factors that induce DNA or RNA viruses. The antimicrobial mechanism is the ability to accumulate inside cells or penetrate phospholipid cell membranes and cause inhibition [23]. The obtained results are consistent with the research results presented by Idris et al. [17]. In their work, these researchers examined the effects of extracts from the leaves and roots of *Rumex crispus L*. on microorganisms and parasites, including *S*. *aureus*, *P*. *aeruginosa* and *E*. *coli*, and found that the antimicrobial activity of the extracts is not dependent on whether the bacteria are Gram-negative or Gram-positive. Orbán-Gyapai [39] showed that curly sorrel root extracts have higher antibacterial effectiveness than leaf extracts. Interestingly, in the case of antifungal activity, the observations were the opposite. Extracts from the fruits of *R*. *crispus L*. also showed good inhibitory activity against pathogenic staphylococci *S*. *aureus* and opportunistic *S*. *epidermidis*, as well as against some Gram-negative bacteria, such as *E*. *coli*, but to a lesser extent [14]. In the case of the antibacterial effect of hops, the literature reports higher effectiveness against Gram-positive bacteria, especially *Staphylococcus aureus*, and lower effectiveness against Gram-negative ones [40]. The low effect of hop-enriched composites was visible in the case of *E*. *coli*, which may be due to the structure of the outer cell membrane of Gram-negative bacteria consisting of lipopolysaccharides, which act as a barrier. However, it is difficult to determine the effect of these composites on bacteria depending on the structure of these microorganisms because, unlike *E*. *coli*, inhibition of the growth of *P*. *aerugino*sa was observed. This may be due to the form of hops found in the composites. The data presented in the literature refer to the biocidal effectiveness of hop extracts or oils, which undoubtedly increases the concentration of killing ingredients in the liquid preparation and their availability for microorganisms.

Comparing the results of our own research and those described in the literature, it can be concluded that both the process of producing oil composites and the composition of the matrix in which natural additives are distributed do not inhibit the biocidal properties of these materials. Attention should also be paid to the possibility of creating biocidal compounds during the composites production process. Natural ingredients added to the mixture of WCO, acid catalyst and sand may degrade [41, 42] as a result of many hours of thermal treatment leading to solidification of the composites, but the final composition of the composites provides them with biocidal properties.

## Conclusions

Oil composites from waste cooking oil (WCO) gain attention in various areas, including in antibacterial studies, due to their sustainable potential and profitable applications. The use of WCO composites in antibacterial and anti-fungal applications is a way to recycle waste products while dealing with the growing problem of bacterial and fungal resistance to traditional antibiotics.

Our research uses two modifiers, curly sorrel and hops, to achieve antimicrobial activity in composite materials. The biocidal effectiveness of enriched composites against Gram-positive bacteria (*S*. *aureus* and *S*. *epidermidis*), Gram-negative bacteria (*E*. *coli* and *P*. *aeruginosa*), and fungi (*A*. *niger*, *P*. *anomala*) has been confirmed. Our research confirms the biocidal effectiveness of oil composites enriched with natural additives, resulting from the antimicrobial properties of hops and curly sorrel or substances resulting from the decomposition of natural additives in the process of creating solid materials or the synergy of all composite components. The results of the conducted research encourage further modification of composites in order to increase the spectrum of their applications. However, the restrictions resulting from the availability of the raw material itself, which is waste food related to the geopolitical situation, should be taken into account.
